# Supplementary material for: Absolute quantification of translational regulation and burden using combined sequencing approaches
Source: Mol Syst Biol. 2019 May 3;15(5):e8719. doi: 10.15252/msb.20188719 (PMC6498945; doi:10.15252/msb.20188719)
Supplement: Supplementary file 1 — Appendix [file MSB-15-e8719-s001.pdf]

Appendix for:

Absolute quantification of translational regulation and burden using combined sequencing approaches

Thomas E. Gorochowski, Irina Chelysheva, Mette Eriksen, Priyanka Nair, Steen Pedersen, Zoya Ignatova

Supplementary Figures **Page**

Figure S1: Expression of the RNA spike-in standards in the RNA-Seq libraries 2

Figure S2: Sequencing data exhibit a good correlation between biological replicates 3

Figure S3: Comparison of ribosome protected fragments (RPFs) mapping to first and second half of each coding region 4

Figure S4: Normalized ribosome P site occupancies across the genome 5

Figure S5: Distributions of mRNA copy numbers and normalized RPF reads 6

Figure S6: Estimating ribosome P site position from an RPF read 7

Supplementary Tables

Table S1: Measurements of cellular parameters 8

Table S2: Sequencing and read mapping statistics 9

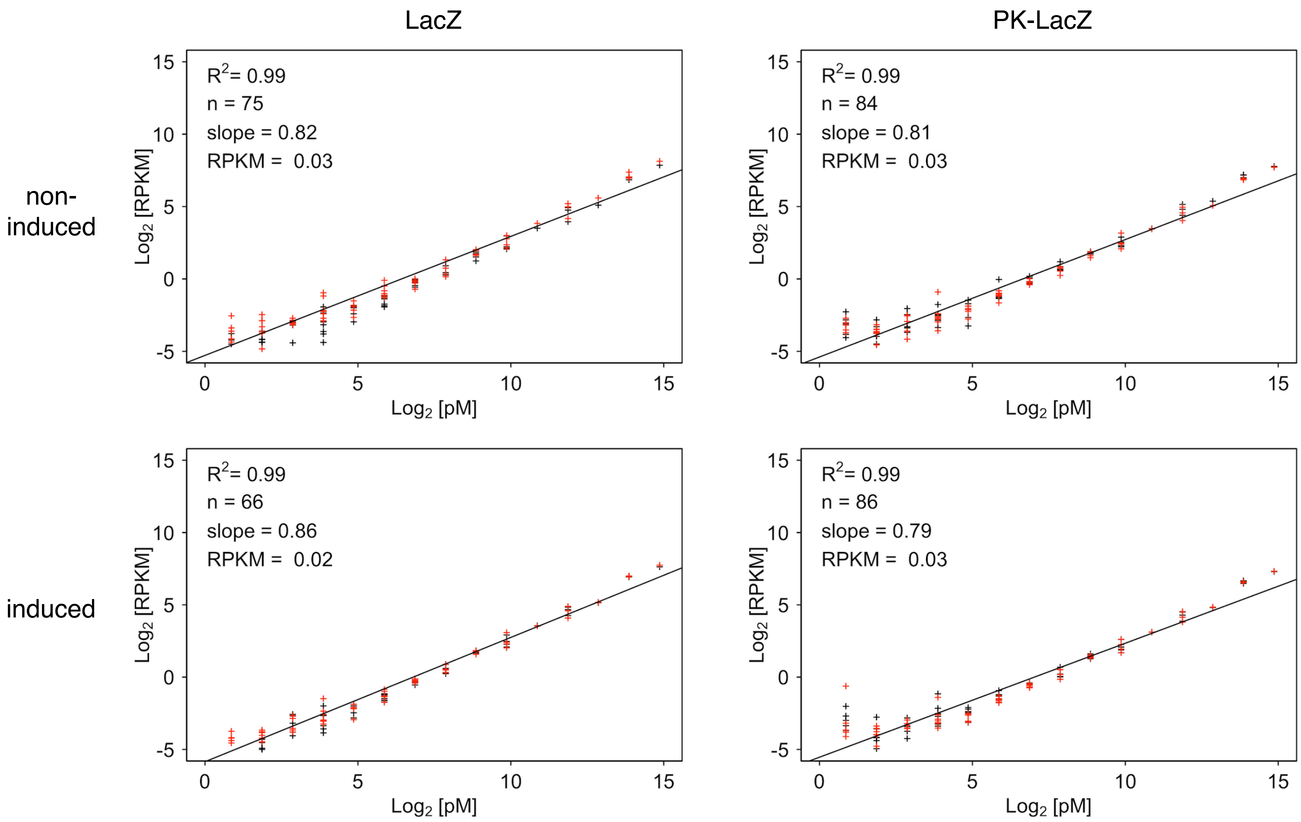

Figure S1: Expression of the RNA spike-in standards in the RNA-Seq libraries. Each point represents a single RNA from the spike-in mixture. Both biological replicates are shown in red and black, respectively. Expression of each spike-in RNA is given in Reads Per Kilobase of transcript per Million mapped reads (RPKM) units. This RPKM value in the inset sets the detection threshold in each RNA-Seq set (**Materials and Methods**). 'n' denotes the number of RNA standards with linear dependence of their concentration in the spike-in mixture (slope); those standards were further considered for copy number determination. R^2, Pearson correlation coefficient.

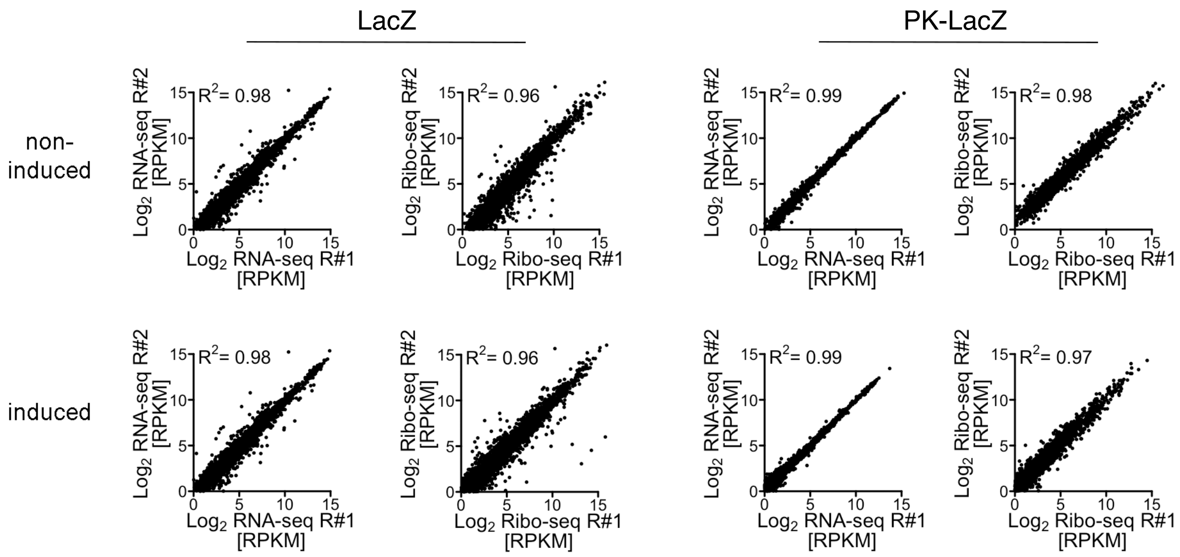

Figure S2: Sequencing data exhibit a good correlation between biological replicates. Correlation of the RNA-seq and Ribo-seq data of two biological replicates from induced and non-induced cells expressing either LacZ or LacZ-PK. Expression of each gene for both RNA-seq and Ribo-seq data sets given in Reads Per Kilobase of transcript per Million mapped reads (RPKM) units. R^2, Pearson correlation coefficient.

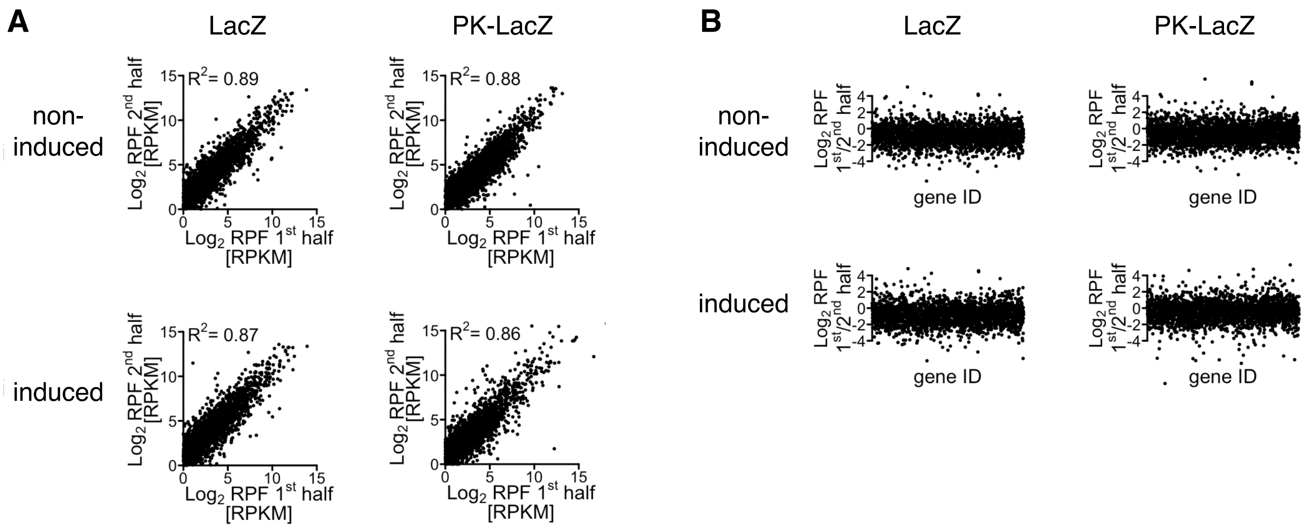

Figure S3: Comparison of ribosome protected fragments (RPFs) mapping to first and second half of each coding region. (**A**) Each point represents a coding region with expression given in Reads Per Kilobase of transcript per Million mapped reads (RPKM) units. (**B**) Log_2 fold-change in RPF counts between 1st and 2nd half of each coding region. Genes ordered alphabetically by name (gene ID). First 51 bp and last 9 bp of each coding region not included in the calculation. R^2, Pearson correlation coefficient.

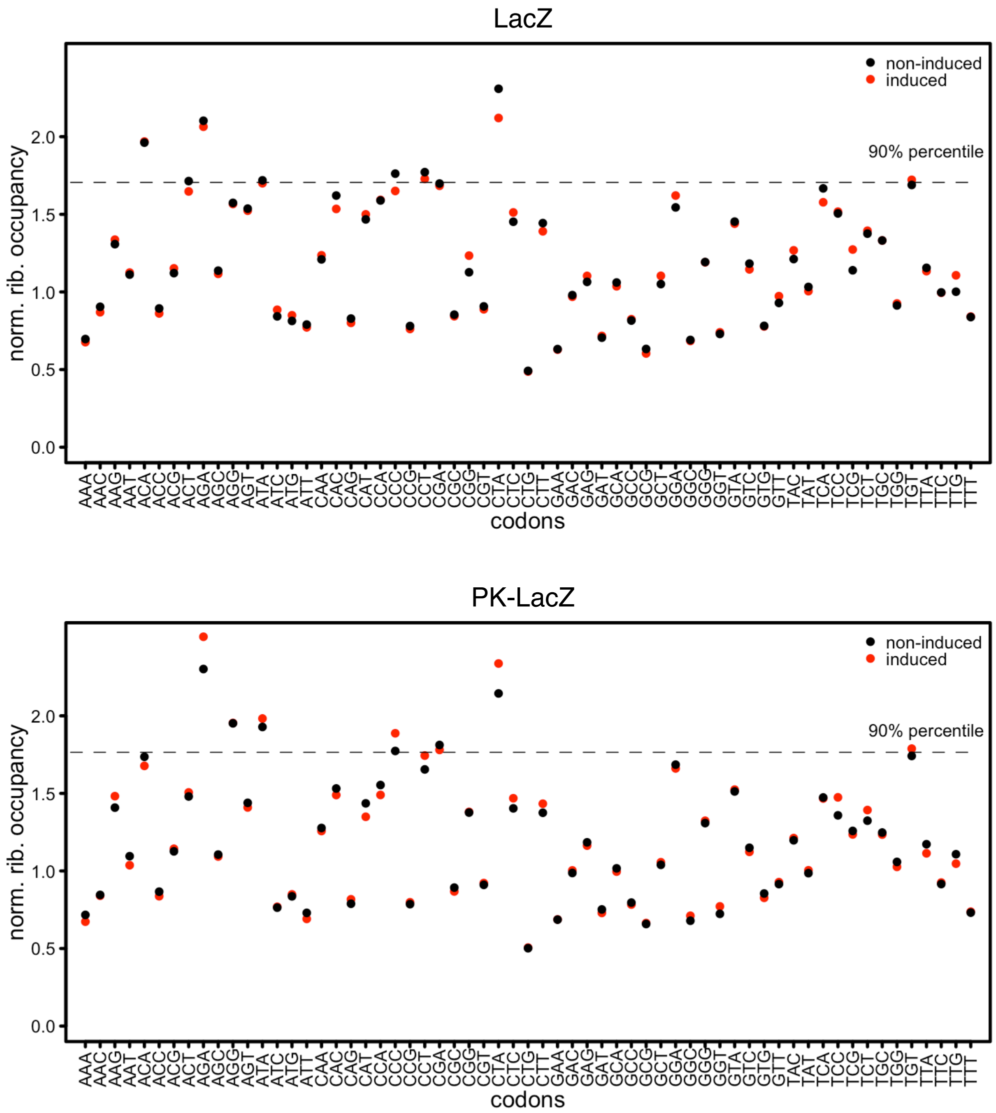

Figure S4: Normalized ribosome P site occupancies across the transcriptome. Values given for each codon for cells harboring LacZ or LacZ-PK variants in non-induced (black) and induced (red) conditions.

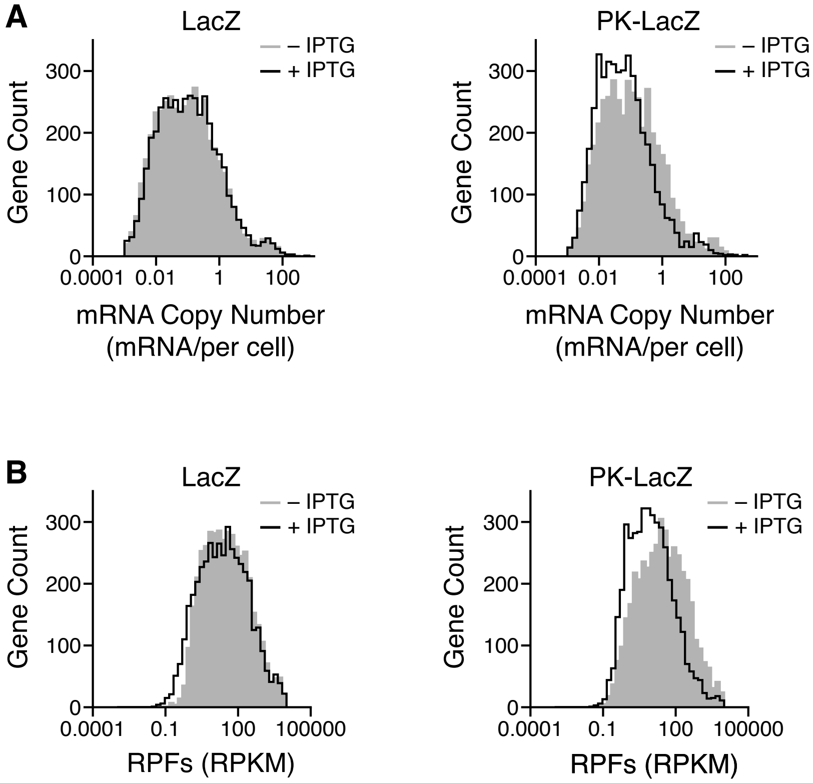

Figure S5: Distributions of mRNA copy numbers and normalized RPF reads. Data shown for cells harboring the LacZ and PK-LacZ variants before and after induction with IPTG (1 mM).

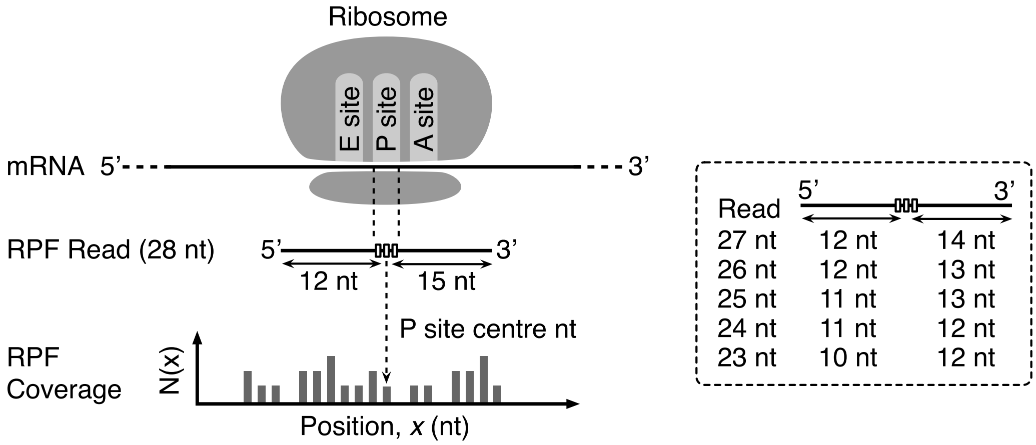

Figure S6: Estimating ribosome P site position from an RPF read. Box shows different lengths used from 5' and 3'-end of various RPF read lengths to calculate position of central nucleotide in the ribosome P site codon.

Table S1: Measurements of cellular parameters

Measurement	Start of induction LacZ	PK-LacZ	End of induction[a] LacZ	PK-LacZ
Doubling time (minutes)	85	85	–[b]	–[b]
Cell count	1.6×10^{9}	1.6×10^{9}	1.6×10^{9}	1.6×10^{9}
Protein mass (grams/cell)	4.13×10^{-13}	4.10×10^{-13}	4.70×10^{-13}	4.30×10^{-13}
Mass of LacZ/PK-LacZ as total of cell	–	–	14%	5.8%

a. Induction of constructs lasted 10 min for LacZ and 15 min for PK-LacZ before samples were taken.
b. No measurable growth detected during the induction period.

Table S2: Sequencing and read mapping statistics

Sample[a]				Total # of reads[b]	Multi-mapped reads[c]	Non-coding[c]	Uniquely mapped to mRNA[c]
PK-LacZ	RNA-seq	non-induced	R#1	66066771	29796556 (45.10%)	2876188 (4.35%)	33394027 (50.55%)
PK-LacZ	RNA-seq	non-induced	R#2	39198111	18025740 (45.99%)	1673252 (4.27%)	19499119 (49.75%)
PK-LacZ	RNA-seq	induced	R#1	70004102	28545341 (40.78%)	1982506 (2.83%)	39476255 (56.39%)
PK-LacZ	RNA-seq	induced	R#2	68146152	27782049 (40.77%)	1931069 (2.83%)	38433034 (56.40%)
PK-LacZ	Ribo-seq	non-induced	R#1	59384989	25736660 (43.34%)	3034480 (5.11%)	30613849 (51.55%)
PK-LacZ	Ribo-seq	non-induced	R#2	62798001	23567687 (37.53%)	6009235 (9.57%)	33221079 (52.90%)
PK-LacZ	Ribo-seq	induced	R#1	56568122	27566889 (48.73%)	2387216 (4.22%)	26614017 (47.05%)
PK-LacZ	Ribo-seq	induced	R#2	54581989	20143633 (36.91%)	3541469 (6.49%)	30896887 (56.61%
LacZ	RNA-seq	non-induced	R#1	192586734	90755510 (47.12%)	7758247 (4.03%)	94072977 (48.85%)
LacZ	RNA-seq	non-induced	R#2	53735584	28463851 (52.97%)	3382171 (6.29%)	21889562 (40.74%)
LacZ	RNA-seq	induced	R#1	190867334	91396079 (47.88%)	6916496 (3.62%)	92554759 (48.49%)
LacZ	RNA-seq	induced	R#2	52249590	25660126 (49.11%)	2776669 (5.31%)	23812795 (45.58%)
LacZ	Ribo-seq	non-induced	R#1	183013690	124093781 (67.81%)	11347972 (6.20%)	47571937 (25.99%)
LacZ	Ribo-seq	non-induced	R#2	53330842	25579314 (47.96%)	4072967 (7.64%)	23678561 (44.40%)
LacZ	Ribo-seq	induced	R#1	199707963	120352995 (60.26%)	7716217 (3.86%)	71638751 (35.87%)
LacZ	Ribo-seq	induced	R#2	41870343	31443591 (75.10%)	1843808 (4.40%)	8582944 (20.50%)

a. Biological replicates are denoted “R#1” and “R#2” and “induced” relates to induction of PK-lacZ or LacZ expression with IPTG (1 mM).
b. Reads aligning to more than one position in the genome, including tRNA and rRNA genes, were excluded from the data.
c. Sequencing reads were mapped to the genome of *E. coli* K-12 MG1655 strain.
